# Formative Evaluation to Increase Availability of Healthy Snacks and Beverages in Stores Near Schools in Two Rural Oregon Counties, 2013

**DOI:** 10.5888/pcd12.150252

**Published:** 2015-12-03

**Authors:** Betty T. Izumi, Nancy E. Findholt, Hayley A. Pickus

**Affiliations:** Author Affiliations: Nancy E. Findholt, School of Nursing, Oregon Health and Science University, La Grande, Oregon; Hayley A. Pickus, School of Community Health, Portland State University, Portland, Oregon.

## Abstract

**Introduction:**

Children living in rural areas are at greater risk for obesity than their urban counterparts. Differences in healthy food access may contribute to this disparity. Most healthy food access initiatives target stores in urban areas. We conducted a formative evaluation to increase availability of healthy snacks and beverages in food stores near schools in rural Oregon.

**Methods:**

We assessed availability of healthy snacks and beverages in food stores (n = 15) using the SNACZ (Students Now Advocating to Create Healthy Snacking Zones) checklist and conducted in-depth interviews with food store owners (n = 6). Frequency distributions were computed for SNACZ checklist items, and interview data were analyzed by using applied thematic analysis.

**Results:**

Overall, availability of healthy snacks and beverages in study communities was low. Four interrelated themes regarding store owner perspectives on stocking healthy snacks and beverages emerged from the interviews: customer demand, space constraints, vendor influence, and perishability.

**Conclusion:**

In addition to working with food store owners, efforts to increase availability of healthy snacks and beverages in rural areas should engage young people, food buyers (eg, schools), and vendors as stakeholders for identifying strategies to increase demand for and availability of these items. Further research will be needed to determine which strategies or combinations of strategies are feasible to implement in the study communities.

## Introduction

Children living in rural areas are at greater risk for obesity than their urban counterparts (1–4). Differences in healthy food access may contribute to this disparity (5–7). Supermarkets, which can be scarce in areas of low population density, nearly always have a wider selection of healthy food at low prices than do convenience stores or small grocery stores, which are more common in rural areas (7,8). Likewise, food stores near rural schools carry fewer healthy snacks and beverages than stores near urban schools (5). Because children often visit food stores near their schools (9,10), increasing availability of healthier options in such stores may help to reduce childhood obesity. Most healthy corner store initiatives have targeted stores in urban areas (11,12).

We describe findings that were collected as part of a larger community-based participatory research project to engage young people in promoting healthy snacking in and around their schools in eastern Oregon. The project, SNACZ (Students Now Advocating to Create Healthy Snacking Zones), reaches young people through 4-H clubs; one SNACZ club is established for each study community. The goal of the evaluation was twofold: obtain baseline data on availability of healthy snacks and beverages in stores near rural schools and understand store owner perspectives on stocking these items. The findings will guide food store intervention strategies in the study communities.

## Methods

This study was conducted in 8 communities of fewer than 2,000 residents in 2 eastern Oregon counties. Five communities were in Union County, the intervention site for SNACZ; 3 were in Wallowa County, the control site. The communities are geographically isolated by mountains or agricultural land. Each community has its own school district, with centrally located elementary/middle and high schools and at least 1 small store, but no supermarkets. In both counties, there are seasonal farm stands, farmers markets, and “u-pick” farms. Approximately 47.9% and 47.3% of students in Union and Wallowa counties, respectively, were eligible for free or reduced-price lunch (13).

This mixed methods convergent study (14) used food store checklists and in-depth interviews. In August 2012, we identified food stores in the 8 study communities by using the telephone book and a database from the Oregon Employment Department (15). We categorized each store as one of the following: a supermarket that sold food and other products (eg, clothing), grocery store that sold canned and dried foods and perishable items, convenience store that sold primarily convenience items (eg, processed snacks, ready-to-eat/heat foods), or gas station food mart (ie, convenience store attached to a gas station). Next, using geographic information system (ArcGIS 10.1; ESRI), we identified stores (n = 15) that fell within a 0.5-mile radial buffer from the geometric center of elementary/middle schools and used a ground-truthing procedure (16,17) to confirm the existence of the stores. We then sent letters to store owners inviting them to participate in SNACZ. One owner of a store in an intervention site community declined the invitation. We therefore identified a second store to work with the SNACZ club in this community. This store fell within a 2-mile radial buffer from the geometric center of the elementary/middle school but was still within the school district boundary. The owner of this store agreed to participate in SNACZ in May 2013. The store owners received a $20 participation incentive. Across the 15 stores, 9 were in the intervention site (2 grocery stores, 5 convenience stores, and 2 gas station food marts); 6 were in the control site (3 grocery stores, 1 convenience store, and 2 gas station food marts). Of the 15 store owners, all agreed to participate in the food store checklists, for a participation rate of 100%. Owners of grocery stores and convenience stores in the intervention site were invited to participate in interviews; 1 convenience store owner declined to participate. The interview participation rate was 86%.

### Assessment of healthy snack and beverage availability

We completed the SNACZ food store checklist (18) during May and June 2013. The checklist included 48 items: 6 beverages, 18 processed snacks, and 24 ready-to-eat and single-portion fresh fruits and vegetables. A “healthy” snack or beverage appropriate for consumption by children was defined as a single-serving product that met the Institute of Medicine Tier 1 nutrition standards for competitive foods and beverages sold in schools for total calories, total calories from fat and saturated fat, *trans* fat, total sugars, and sodium (19). We also assessed availability of the same items in multiportion sizes. For each of the 4 categories of items, multiportion was defined as follows: beverages in portion sizes greater than 8 ounces; processed snacks with more than 1 serving per package; fresh produce sold in bags, bundles, party trays, or large containers (eg, strawberries in quart-size container). The checklist was completed by a trained research assistant. The checklist took approximately 8 minutes to complete. All SNACZ study procedures were approved by the Oregon Health and Science University Institutional Review Board, Portland, Oregon.

We pooled checklist data from stores in the intervention and control sites for this analysis to increase the sample size. We computed frequency distributions for individual food and beverage items by using Stata software (version 11; StataCorp LP).

### Food store owner interviews

We conducted the interviews during May and June 2013. The semistructured interview guide included questions about ordering snacks and beverages; experience selling healthy snacks and beverages, including fresh produce; and barriers to stocking healthy snacks and beverages (Box). The interviews lasted approximately 45 minutes and took place in the owners’ stores. The interviews were audio-recorded and transcribed verbatim.

**Box. Questions for interviews with owners of food stores in a rural Oregon county**

- When you are ordering snack foods, what do you consider? Do you try to carry a selection of healthier snacks, such as baked chips or low-fat baked goods like bagels?
- If you currently carry, or have tried to carry, healthier snacks in your store, what has been your experience with selling these?
- When you are ordering beverages, what do you consider? Do you try to carry a selection of healthier beverages, such as 100% fruit juice or skim milk?
- If you currently carry, or have tried to carry, healthier beverages in your store, what has been your experience with selling these?
- What do you consider when ordering fruits and vegetables for your store?
- Do you carry, or have you tried to carry, any ready-to-eat fruits or vegetables that children might choose as snacks? If yes, what has been your experience with selling these?
- What do you see as being the major barriers to carrying healthier snacks and beverages?
- What, if anything, could potentially be done to overcome these barriers?

We analyzed the interview data by using applied thematic analysis (20,21) targeted toward discovering themes with practical applications. We created a codebook with 13 operationally defined codes based on the interview questions and preliminary review of interview transcripts. Authors B.T.I. and N.E.F independently coded all 6 interview transcripts and compared them for inter-coder reliability. We resolved coding discrepancies through discussion and clarifying code definitions until we reached 100% agreement. When major code changes were made, we recoded the data with a revised dictionary. After all interviews were coded, we developed a series of displays (21) to determine themes within and across interviews and to draw and verify conclusions about the data.

## Results

### Healthy snack and beverage availability

Overall, few stores carried healthy snacks or beverages in multiportion sizes; even fewer carried the checklist items in single-portion sizes (Table 1). All stores carried plain bottled water in both sizes; other healthy beverages (ie, low-fat and nonfat plain milk, flavored milk, 100% fruit juice, soy milk) were available only in a multiportion size. All stores carried single-portion nuts and seeds and most carried multiportion crackers, nuts and seeds, graham or animal crackers, and dried fruit that met the checklist criteria. Six of 18 processed snacks were not available in any store in any size. Only one-third of the stores carried yogurt in a single-serving container that met the checklist criteria. Across the stores, availability of fresh produce in both single-portion and multiportion sizes was low. Most stores sold loose apples and oranges but fewer than half carried loose bananas; other ready-to-eat fruits were uncommon. Availability of fresh vegetables in single-portion sizes was rare; across the stores, cherry tomatoes were the only single-portion product available (n = 4). The number of stores that carried fresh produce in multiportion sizes also was low. Five stores carried strawberries in quart-size containers and about one-quarter carried bananas and grapes in large bunches. Only 6 stores carried baby carrots in multiportion bags. Bags of apples were not available at any store, and only 2 carried bags of oranges.

**Table 1:** Availability of Healthy Snacks and Beverages in Single-Portion and Multiportion Sizes in 15 Stores Near Rural Elementary/Middle Schools, Oregon, 2013

Category/Item	Single-portion, % (n)	Multiportion, % (n)
**Beverages**		
Water without flavoring, additives, carbonation, or caffeine	100 (15)	100 (15)
Low-fat (1%) milk, 8-oz portion	0	67 (10)
Nonfat milk, 8-oz portion	0	60 (9)
1% or nonfat flavored milk, 8-oz portion	0	7 (1)
100% fruit juice	0	100 (15)
Soy milk	0	33 (5)
**Snacks**		
Chips	0	0
Chex Mix	0	60 (9)
Crackers	0	80 (12)
Pretzels	0	7 (1)
Rice cakes	0	27 (4)
Popcorn	0	0
Nuts and seeds	100 (15)	93 (14)
Trail mix	0	0
Cookies	0	47 (7)
Graham or animal crackers	0	93 (13)
Granola bars	33 (5)	53 (8)
Bagels	0	0
Muffins	0	0
Popsicles or other frozen desserts	0	0
Yogurt	33 (5)	13 (2)
Applesauce, unsweetened	0	33 (5)
Other canned or bottled fruit	0	60 (9)
Dried fruit with no added sugar	0	73 (11)
**Fresh fruits**		
Apples	60 (9)	0
Apricots	7 (1)	0
Bananas	47 (7)	27 (4)
Blueberries	13 (2)	0
Cherries	0	20 (3)
Grapefruit	20 (3)	0
Grapes	7 (1)	27 (4)
Melon (cut up)	0	13 (2)
Nectarines	7 (1)	0
Oranges	60 (9)	13 (2)
Peaches	13 (2)	0
Pears	33 (5)	0
Pineapple (cut up)	0	7 (1)
Plums	0	0
Strawberries	0	33 (5)
Mixed fresh fruit (ie, fruit salad)	0	13 (2)
Other ready-to-eat and single-portion fresh fruit (eg, kiwis, figs)	33 (5)	0
**Fresh vegetables**		
Broccoli florets	0	7 (1)
Carrots (baby)	0	40 (6)
Cauliflower florets	0	7 (1)
Celery sticks	0	0
Cherry tomatoes	27 (4)	7 (1)
Mixed fresh vegetables	0	7 (1)
Other ready-to-eat and single-portion fresh vegetables (eg, snap peas)	0	20 (3)

### Food store owner interviews

Four interrelated themes regarding store owner perspectives on stocking healthy snacks and beverages emerged from the interviews: customer demand, space constraints, vendor influence, and perishability (Table 2).

**Table 2:** Themes and Supporting Quotes From Qualitative Interviews With Store Owners (n = 6) in a Rural Oregon County, 2013

Theme	No. of Store Owners Referencing Theme	Quotes to Support Theme
**Customer demand**	6	“We try to stay customer-oriented. That’s the main thing. I want my customers coming through the door…that’s the main drive of anything. You want to bring in something that’s going to sell, that’s not going to sit there and be dead inventory.” (Store 11: grocery store)
		“I would love to carry celery sticks and carrots and ranch dip and stuff, but I tried it and they do not sell. I can’t keep buying that stuff and throwing it away.” (Store 14: convenience store)
		“We sell a lot of yogurt. That has surprised me. . . . I’ve noticed a lot of kids getting a yogurt with their meal. I think they learned that at school with some programs. Maybe even starting in preschool. They get started in school and then they like it.” (Store 1: grocery store)
**Space constraints**	5	“We just don’t have very much room for [healthier products].” (Store 1: grocery store)
		I have a small area, so I have to take [that] into consideration. If [healthy products are] requested and I have a place to market them, I would do that. (Store 15: convenience store)
**Vendor influence**	6	“The contract we have [with vendors] indicates that vendors will take back the expired products, so vendors don’t want us to stock what they believe won’t sell.” (Store 10: convenience store)
		“[M]y soda coolers are provided by the distributors, so I have to carry in it what they distribute. . . . Most of [the vendors] merchandise the accounts, so they put in and kind of control the inventory.” (Store 15: convenience store)
		“With fresh fruits and vegetables, sometimes they are really hard to get in. So, I will run to [larger community] so we have it in here. Because it’s too expensive for us to get it trucked here and be able to carry it at a reasonable price for children or young adults who may want to purchase them.” (Store 13: convenience store)
**Perishability**	4	“I am fortunate because I have the restaurant so I can rotate stuff over before it goes bad. The items I can use, [like] the produce, I can take it over and make soups out of it. I have a benefit that other stores don’t have.” (Store 14: convenience store)

#### Customer demand

Across the 6 store owners, customer demand was the primary criterion by which they made their stocking decisions. One participant said, “If I think it is something that will sell in my location I may initially order some of it to try and see if it sells and that determines whether I will keep it in stock or not” (Store 15: convenience store).

The participants indicated that, although they did stock some healthy snacks and beverages, the items were largely selected based on customer requests. For example, 3 store owners indicated that they kept low-sugar and gluten-free items in stock to meet the needs of their customers with diabetes or who are gluten intolerant. Five store owners indicated that they have tried to carry healthy snacks and beverages in their stores and that their experiences were generally unsuccessful: sales of packaged snacks (eg, baked chips) “come and go” but were not fast movers, and fruits and vegetables, except for apples and oranges, often spoiled before they sold.

#### Space constraints

Five of 6 store owners indicated that space constraints limited the number and variety of products they could carry in their stores. Store owners were reluctant to devote scarce shelf space to new products and instead preferred to stock products that had proven records of selling. In addition, because many products were available only through their vendors in large case-pack quantities and because maintaining items in stock is costly, store owners were hesitant to try new products, especially perishable products that may not have a high turnover (ie, the number of times inventory is sold and replaced during a certain time period). For example, 1 participant said, “With fruit, we’re not able to carry a variety. For example, we can’t carry 2 or 3 different kinds of apples because we need to buy them in 40 lb. boxes so we can only buy one variety” (Store 10: convenience store).

#### Vendor influence

All store owners reported that their vendors carried few healthy snacks and beverages. When healthier options were available, they were generally available only in large quantities (eg, 24 bags of baked potato chips, 40 lb box of apples), too large to sell to store owners’ limited customer base. Those who wanted to carry these products purchased small quantities “in town,” and re-sold them at their stores. The driving distance for these “buying trips,” which typically took place at stores in the larger surrounding communities, was approximately 50 to 80 miles round-trip. One store owner said that he was able to work with his vendor to purchase small quantities of products on a case-by-case basis. Four store owners also indicated that vendor “buy-back” agreements, in which vendors repurchased products that didn’t sell, also influenced what they carried. Although these agreements reduced the financial risk of trying new products for store owners, they increased the financial risk for vendors, which made vendors wary of carrying products they perceived to be slow movers. For example, 1 participant said

[This practice] is one reason why they are so leery about bringing in an item. If they have to buy it back outdated, they lose on their commissions. . . . [I]f they brought in 100 loaves of bread for me and they have to buy back 20, they have to take that off their commission. They are very leery about bringing in new items that they don’t think will sell. (Store 11: grocery store).

Vendors also promoted specific products to store owners, which influenced what the stores carried. One participant said

Most of our beverages come from Pepsi and Coke. . . . [Our vendors say] “This juice is really hittin’ it right now, so can we pull this product and put this in instead?” And I like trying this . . . so, if we have a suggestion from them, it seems that we always go with it. (Store 13: convenience store).

For some products, vendors provided the food and beverage displays (eg, coolers) and controlled the display inventory.

#### Perishability

When asked to identify barriers to stocking healthy snacks and beverages, 4 of 6 participants indicated that perishability of products such as fresh fruits and vegetables made it difficult to carry these items. One participant said

I think the main thing is the healthier something usually is, the shorter the lifespan is on the shelf. That’s the big thing. You’ve got less preservatives, less holding power. The short lifespan is probably the biggest thing, the hardest thing for me. . . . If I had unquestionable amount of time, it wouldn’t bother me to let it sit there. But once it goes outdated, I have to mark it down or get rid of it and that makes it cost prohibitive. (Store 11: grocery store).

Store owners did state that because demand for fruits and vegetables was higher during the summer they were more likely to stock a wider variety of these items, but only on a seasonal basis. Other perishable products carried by some of the store owners included those they were required to stock as a store authorized to accept payment through the Special Supplemental Nutrition Program for Women, Infants, and Children. Two participants had an in-store deli or an adjacent restaurant, in which they repurposed perishables (eg, day old bread, bananas with brown spots) as ingredients in their deli or restaurant menu items (eg, croutons, banana bread) before the products spoiled. This practice for reducing shrink (ie, the difference between what can potentially sell at full retail minus what is actually sold) by repurposing food is common in the food service industry.

## Discussion

Consistent with previous studies of small stores in both urban and rural areas (5,6,22–24), most items on the SNACZ food store checklist were not available or found only in a small percentage of the stores surveyed. Our findings, and those of others (24–26), suggest that low availability of healthy options in small stores is due, in part, to a lack of perceived customer demand for these products. Because the stores in our study are near schools, engaging young people may be an effective strategy for creating and increasing demand for specific snacks and beverages that can be sold in the participating food stores. In Philadelphia, the Food Trust has worked with young people to increase the supply of healthy options in food stores near urban schools and the demand for these products by young people (27).

Our work also suggests that in rural areas, low population density and lack of product delivery options may also contribute to low availability of healthy options. Consistent with findings reported by others (28,29), store owners in our study indicated that when healthy snacks and beverages were available through their vendors, they were available only in quantities too large for their limited customer base. Although 1 store owner indicated that his vendor was willing to work with him to meet his needs, others indicated that their vendors were unwilling to split cases. Because of their location, the store owners in our study were able to select from only a limited number of vendors, which further hampered their ability to stock healthy options, including produce.

Given the challenges described above, potential strategies for increasing availability of heathy options in food stores participating in SNACZ include establishing partnerships between food stores, schools, and SNACZ clubs to develop consistent marketing messages to increase demand for healthy snacks and beverages that can be purchased in the food stores; providing store owners with financial incentives to offset costs associated with increasing availability of healthy snacks and beverages (eg, cooler to display single-portion fruit); facilitating partnerships between food stores and other food buyers (eg, schools) to coordinate their orders to increase their respective purchasing power; and engaging vendors as stakeholders in strategies to increase availability of healthy snacks and beverages, especially in single-portion sizes, in food stores.

Strengths of this study include our mixed-methods approach, which allowed us to illustrate checklist results with interview findings to develop a more complete understanding of healthy snack and beverage access in the study communities. Limitations include the small sample size, the potential for interview participant response bias, and the narrow geographic location, which limits generalizability of our findings to other rural areas.

Convenience stores and small grocery stores may be important sources of healthy snacks and beverages for children in rural communities, where availability of affordable and healthy food can be scarce. Efforts to increase availability of healthy snacks and beverages in these communities should engage young people, food buyers (eg, stores, schools), vendors, and store owners. Further research is needed to determine which strategies are feasible to implement in the study communities.
